# 

**Published:** 1875-06

**Authors:** 


### Books and Pamphlets Received.

---

Cyclopædia of the Practice of Medicine. Edited by Dr. H. Von Ziemssen, Vol. III, Chronic Infectious Diseases. American Translation. Edited by Albert H. Buck, M. D. New York: Wm. Wood & Co., 1875.

Transactions of the Ninth Annual Meeting of the Medical Association of Missouri, April 20th and 21st, 1875.

Medical Address. By Benjamin E. Cotling, A. M., M. D. Boston: David Clapp & Son, 1875.

Treatment of Uterine Displacements. By H. F. Campbell, M. D., Augusta, Ga. From Atlanta Medical and Surgical Journal.

A Case of Reflex Neuralgia, associated with Urectral Contractions. By F. N. Otis, M. D. From New York Medical Journal.

### Medical and Surgical Journal

PUBLISHED MONTHLY,

Containing Original Articles, Reports of Medical Societies and Hospitals, Editorial, Reviews, Correspondence, Army News, etc., etc; including the usual variety of Medical Periodical Publications. Specimen copies sent on application

Address Buffalo Medical & Surgical Journal, Buffalo, N. Y.

TERMS OF SUBSCRIPTION, - - $3 0) PER YEAR, IN ADVANCE.

